# Atomistic simulation of the measurement of mechanical properties of gold nanorods by AFM

**DOI:** 10.1038/s41598-017-16460-9

**Published:** 2017-11-24

**Authors:** Bernhard Reischl, Andrew L. Rohl, Antti Kuronen, Kai Nordlund

**Affiliations:** 1 Curtin Institute for Computation and Department of Chemistry, Curtin University, GPO Box U1987, Perth, WA 6845 Australia; 2 Department of Physics, University of Helsinki, PO Box 43, Helsinki, FI-00014 Finland

## Abstract

Mechanical properties of nanoscale objects can be measured with an atomic force microscope (AFM) tip. However, the continuum models typically used to relate the force measured at a certain indentation depth to quantities such as the elastic modulus, may not be valid at such small scales, where the details of atomistic processes need to be taken into account. On the other hand, molecular dynamics (MD) simulations of nanoindentation, which can offer understanding at an atomistic level, are often performed on systems much smaller than the ones studied experimentally. Here, we present large scale MD simulations of the nanoindentation of single crystal and penta-twinned gold nanorod samples on a silicon substrate, with a spherical diamond AFM tip apex. Both the sample and tip sizes and geometries match commercially available products, potentially linking simulation and experiment. Different deformation mechanisms, involving the creation, migration and annihilation of dislocations are observed depending on the nanorod crystallographic structure and orientation. Using the Oliver-Pharr method, the Young’s moduli of the (100) terminated and (110) terminated single crystal nanorods, and the penta-twinned nanorod, have been determined to be 103 ± 2, 140 ± 4 and 108 ± 2 GPa, respectively, which is in good agreement with bending experiments performed on nanowires.

## Introduction

At the nanoscale, mechanical properties can deviate from those of the ‘bulk’ material and often exhibit a strong dependence on the object’s size and shape^1–3^. Indentation experiments and hardness tests are typically carried out with nanoindenters using a tip with a well-defined geometry^4^. However, even the smallest accessible forces (~1 mN) and indentation depths (~10 nm) can be too large to study a true nanoscale object. Also, it is not trivial to even locate a nanoscale sample on a substrate, and position the nanoindenter on top of it. Atomic Force Microscopes (AFM) can be used to locate and image these nanoscale objects as well as indent the sample with the required force sensitivity. Provided the cantilever’s spring constant has been well calibrated, a proper force-distance curve for the indentation can also be obtained. This technology is already well established in the study of elastic properties of softer biological tissues^5^ and even single cells^6^. More recently, it has also been used for indentation on solid nanoscale objects such as nanoparticles and nanowires^2,7–10^.

In addition to the technical challenges of these nanoscale experiments, it is not straightforward to calculate mechanical properties, such as stiffness or Young’s modulus, from the measurement. Continuum models are assumed to describe contact mechanics between two objects with a certain geometry^11^, in order to relate force and indentation depth to a mechanical characteristic of the object. These models are pushed to the limits of their applicability when indenting nanoscale objects, as they exhibit large surface-to-volume ratios and atomic scale roughness^12^. Computer simulations, however, can provide insight on the underlying atomic scale processes which are not captured in continuum or finite element models. Atomistic simulations have been used to study indentation^13–16^, bending^17^, tensile load^18^ or sliding friction^19^ in various systems.

In this article, we present simulations of AFM nanoindentation of single crystal and penta-twinned gold nanorods on a silicon (100) substrate, at the actual scale of commercially available nanorods and diamond AFM tips. The mechanical properties of gold nanorods have been shown to depend on their size and shape^3^ and it is experimentally possible to synthesise nanowires or nanorods with specific aspect ratio and crystallographic structure^20–22^. While it is straightforward to simulate a nanoscale object’s response to uniaxial compression, the aim of this study is to model the actual experimental setup as closely as possible. This allows us to investigate the atomistic details of incipient plasticity and dislocation dynamics in the three different samples under load. By applying the Oliver-Pharr method^23,24^ to the unloading force-distance curves from the simulation, we can calculate the Young’s moduli of the samples in exactly the same way as in experiment, thereby enabling a direct comparison.

### Simulation details

We used large-scale molecular dynamics calculations (MD) to simulate the nanoindentation of three systems consisting of a gold nanorod on a silicon slab with a semi-spherical diamond AFM tip apex. The simulation protocol was similar to the one employed in previous work^16^. In brief, the tip model was initially placed 0.5 nm above the centre of the nanorod. At the beginning of each simulation, the tip was rigidly shifted by Δ*z* = −0.025 nm towards the sample, followed by 10 ps equilibration, and then the force along *z* on the tip atoms was sampled over 40 ps. This procedure was iterated until an indentation depth of 2.7 nm, or 10% of the nanorod diameter, was reached. Then the tip was retracted, using the same protocol, until the force on the tip vanished.

The AFM tip apex was modelled after diamond tips from SCD Probe (D300 series, nominal tip radius 10 nm), which may be used for nanoindendation as well as imaging in dynamic mode AFM. A semi-sphere of radius *r* = 10 nm was cut out of bulk diamond with the [111] direction aligned with the direction of indentation. All carbon atoms in the tip were ‘frozen’ to the initial relaxed geometry, as previous studies had shown that no significant deformations or reconstructions occur at the diamond tip apex during indentation^16^, allowing more efficient sampling of the force on the tip.

The gold nanorods were modelled after commercial samples from Nanopartz (Bare Gold Nanorodz, item nr. 30-25-600). In order to study the effect of crystallographic structure and orientation of the sample, we considered two nanorod models in this study. First, a single crystal nanorod with an octagonal cross-section, as described by Wang *et al*.^25^, with the long axis along the [100] direction, that could expose either a (100) or (110) facet to the tip. All corners were capped at 45° by (100), (110) and (111) facets, to lower the surface energy. This geometry was confirmed in 3D reconstructions of high-angle annular dark-field scanning transmission electron microscopy (HAADF-STEM) images^26^. We also considered a penta-twinned nanorod with the main axis along the [110] direction, exposing five (100) facets, and capped on either end by five (111) facets, a geometry that had also been found to be stable from thermodynamic calculations^27^. The commercial samples are likely to have a penta-twinned structure, as they are synthesised according to the method of Murphy *et al*.^28^. Both nanorod models were approximately 55 nm long and had a diameter of 27 nm.

**Figure 1:**
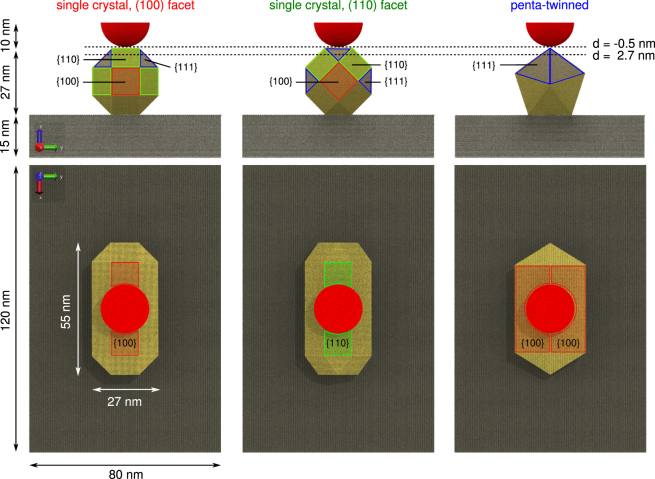
Simulation setup. Systems consist of a spherical diamond tip apex of 10 nm radius (red), the gold nanorod samples (gold), and the silicon slab (grey). The single crystal nanorod with the octagonal cross section can expose either a (100) facet, or a (110) facet, to the tip. The different crystallographic planes defining the surfaces on the nanorods are highlighted in red, green and blue for {100}, {110} and {111} planes respectively. The indentation depth simulated ranged from approximately −0.5 nm to 2.7 nm, in steps of 0.025 nm, as indicated by the two dotted lines.

The nanorods were placed on a silicon (100) slab, measuring $120\times 80\times 15$ nm^3^. Four atomic layers (${\rm{\Delta }}z=0.54$ nm) at the bottom of the slab were frozen to bulk-like positions, and periodic boundary conditions were applied on the simulation cell along the *x* and *y* directions. Having an explicit substrate this large was deemed necessary to study any dislocations appearing in the substrate during the indentation of the nanorod, while avoiding artefacts arising from periodic boundaries. Figure 1 illustrates the three model systems, each containing over 10,000,000 atoms in total. All simulations combined required over 4 million CPU hours on a Cray XC 40 supercomputer.

The atomistic interactions in the system were described by Tersoff-style bond order potentials for Au-Au, C-C, and Si-Si^29,30^. Au-C interactions were described by a Lennard-Jones potential^31^ and Au-Si interactions by a Morse potential^32^, both switched to zero at a distance ${r}_{{\rm{c}}}=0.5$ nm. The PARCAS molecular dynamics code^33^ was used with a time step ${\rm{\Delta }}t=0.71$ fs. This small time step value (0.02 internal time units) was chosen to ensure good energy conservation during the equilibration phase following the initial shift of the tip. To dissipate the heat introduced to the system by the indentation and maintain a temperature $T=300$ K, a Berendsen thermostat^34^ with a time constant $\tau =1$ ps was applied to all moving atoms in the system.

To detect and visualise the dislocations in the systems under load, the final frame of each MD simulation along the indentation and retraction was processed with the analysis tool OVITO^35^. A common neighbour analysis was performed and atoms inside the nanorod exhibiting normal fcc coordination removed. The remaining Au atoms (surface atoms, dislocations, stacking faults and atoms around vacancies) were then color-coded according to their coordination number.

## Results and Discussion

**Figure 2:**
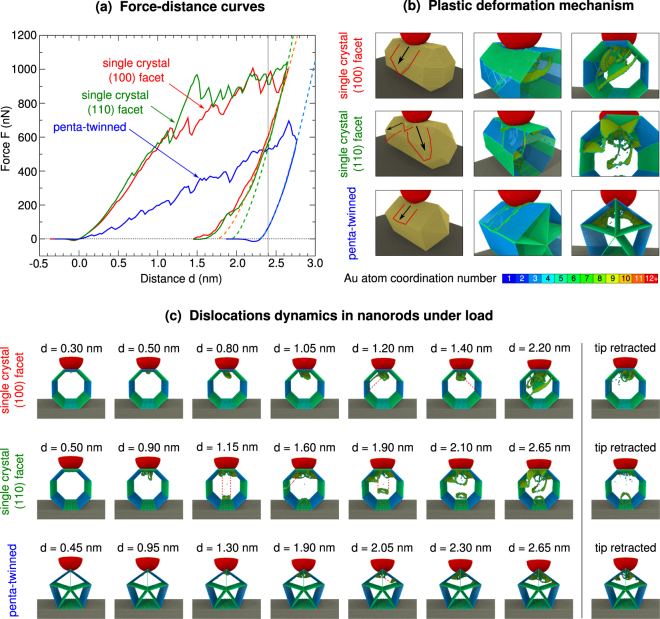
Force-distance curves, dislocation dynamics and plastic deformation under load. (**a**) Force-distance curves along indentation and retraction on the single crystal gold nanorod exposing a (100) or (110) facet to the AFM tip, and the penta-twinned gold nanorod. Fits to the initial part of the unloading curves (*d* > 2.4 nm) are shown as dashed curves. (**b**) Snapshots of the three systems at maximum indentation depth indicate different plastic deformation mechanisms. Red lines highlight the volume of displaced material and black arrows indicate the flow of material. The deformation mechanisms can be understood in terms of the orientation of {111} glide plane systems with respect to the nanorod geometry. (**c**) Snapshots along the indentation trajectories, indicating dislocation nucleation, migration and annihilation at the nanorod surfaces, which are responsible for the discontinuities in the force-distance curves (see supplementary information online for a movie of the dislocation dynamics). Dashed red lines illustrate dislocation loop trajectories. Some dislocations persist in the nanorods even after the tip has been fully retracted.

In Fig. 2a we present the force-distance curves obtained along a full indentation and retraction cycle on the (100) and (110) terminated single crystal nanorods, and the penta-twinned nanorods. The force-distance curves have been shifted to set the distance to zero when the force on the tip first becomes repulsive. The nucleation, propagation and annihilation of dislocations in nanorods under load, responsible for the different plastic deformation mechanisms shown in Fig. 2b, are illustrated by snapshots of the MD simulations in Fig. 2c, but we encourage the reader to also look at the full movies of the indentation simulation, provided as supplementary data.

### Indentation mechanism

In the single crystal nanorods exposing (100) or (110) facets to the AFM tip, the force-distance curves initially have a similar profile. Dislocations under the area of indentation are nucleated at indentation depths $d\le 0.5$ nm, visible as small drops in the force-distance curves. However, due to the different orientation of the fcc lattice with respect to the indentation direction, the dislocations propagate in different ways in the two single crystal samples.

In the nanorod exposing a (100) facet to the tip, dislocations first nucleate at *d* $\simeq$ 0.30 nm. At $d\simeq 0.50$ nm, the characteristic V-shape of two {111} planes appears underneath the area of indentation, accompanied by a small drop in force. In the range $0.50\le d\le 1.20$ nm, more dislocations appear and one of the {111} stacking faults increases in size. At $d\simeq 1.20$ nm, the dislocations can rapidly move to the side of the nanorod and annihilate at the free surface, releasing stress, and leading to a noticeable drop in force. The next big drop in force at $d\simeq 1.40$ nm marks a similar plastic deformation on the other side of the nanorod. At the same time, a partial dislocation loop pointing towards the back of the nanorod is formed, but it cannot reach the free surface during the remainder of the indentation. However, several other dislocations annihilate at the sides of the nanorod when $1.75\le d\le 2.60$ nm. This simple mechanism, where wedges of gold atoms are being moved down the sides of the nanorod, clearly determines the plastic deformation of the (100) terminated nanorods.

The nanorod exposing a (110) facet to the tip exhibits a different deformation mechanism. The initial dislocation nucleation happens at $d\simeq 0.50$ nm. When $0.60\le d\le 1.00$ nm, multiple partial dislocation loops appear under the area of indentation. The first full dislocation loop is emitted towards the bottom of the nanorod when $d\simeq 1.15$ nm. Partial dislocation loops are also pushed to the sides of the nanorod, causing pile up of material around the indenting tip. At $d\simeq 1.60$ nm, partial loops emitted diagonally towards both ends of the nanorod start to annihilate at the surface, causing a large drop in load. This third plastic deformation mechanism requires the dislocations to travel further, compared to the deformation towards the sides of the (100) terminated nanorod, and this is the reason why the forces on the (110) terminated nanorods are significantly larger than on the (100) terminated nanorods for indentation depths $1.15\le d\le 1.60$ nm. At $d\simeq 1.90$ nm, the second dislocation loop is emitted towards the bottom of the nanorod, while the previous one is annihilated at the bottom. A third partial loop appears at $d\simeq 2.10$, but is not emitted during the remainder of the indentation. In the range $1.50\le d\le 2.65$ nm, all three mechanisms continue to occur simultaneously, and the forces remain almost constant around 1000 nN, as stress is released through plastic deformation.

Over the entire indentation distance range, the force on the penta-twinned nanorod remains lower than on the single crystal samples. This can be explained by the fact that the penta-twinned nanorod exposes an edge to the tip and not a flat surface. The first dislocations nucleate at indentation depths of $d\simeq 0.15$ nm on either side of the twinning plane. The first noticeable drop in force occurs at $d\simeq 0.19$ nm when the atoms on the nanorod edge under the tip have been displaced in {111} planes parallel to the twinning plane, and at $d\simeq 0.30$ nm a further drop occurs when atoms are pushed out of the surface plane next to the tip, in a first plastic deformation, annihilating the dislocations. These atoms are pushed back into the nanorod as the tip descends further, and more noticeable dislocations are formed at $d\simeq 0.45$ nm, which also partly annihilate at the free surface, leading to a drop in force around $d\simeq 0.50$ nm. Similar events occur at $d\simeq 0.65$ and 0.85 nm. Then, a more complicated dislocation forms, and a partial dislocation loop is emitted at $d\simeq 0.95$ nm and moves down underneath the surface towards the left side of the nanorod. Around $d\simeq 1.15$ nm, several partial dislocation loops appear on either side of the twinning plane. When $1.20\le d\le 1.80$ nm, new dislocations continue to be nucleated; some of them rapidly move to the free surface and annihilate, while others persist. Around $d\simeq 1.90$ nm, large partial dislocation loops between the vertical twin plane and the free surfaces have appeared, and at $d\simeq 2.05$ nm one of them is gliding down the right side of the nanorod. At $d\simeq 2.30$ nm, both partial loops hit the corner between the free surface and the next twinning planes and get pinned. At deeper indentation depths, more partial loops glide down the sides, but the dislocations are trapped at the twinning plane, and no nucleation of new defects on the other side of the twinning plane is observed, even at the maximum indentation forces of 500 nN.

In summary, both the single crystal and penta-twinned nanorods exhibit only a very short elastic regime, for indentation depths below 0.15–0.5 nm, depending on the sample. Once dislocations have been nucleated, the deformation of the samples under load mainly involves migration of dislocations in $\mathrm{\{111\}}$ glide planes, as expected for an fcc metal. Depending on the crystal structure and orientation of the sample, this leads to different deformation mechanisms of the nanorods under load, as illustrated in Fig. 2b. The dislocation nucleation and emission mechanism (see Fig. 2c) is similar to the one observed in smaller gold nanorods, as well as bulk gold (100) and (110) surfaces^16^. It is also consistent with other simulations of the indentation of bulk metal surfaces using EAM potentials^36^.

### Unloading

The deformation of the nanorods under load, illustrated in Fig. 2b, is irreversible. During tip retraction, the force decreases faster than during the indentation for all three samples and the force-distance curves also exhibit more similarity between them. After the tip has been fully retracted, partial dislocation loops remain under the area of indentation in the (100) terminated nanorod. In the (110) terminated nanorod, a full dislocation loop remains pinned at the bottom of the nanorod. In the penta-twinned nanorod, many dislocations remain as well, including the ones trapped at the twin planes. The dislocations in the three nanorods after tip retraction are shown in Fig. 2c.

### Effect of the substrate

**Figure 3:**
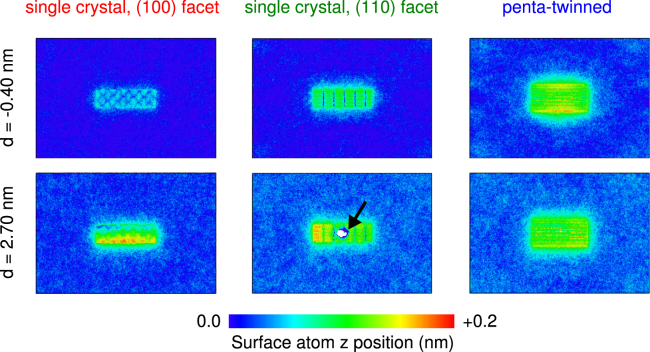
Substrate deformation. Height profile of the silicon substrate before the indentation (*d* = −0.4 nm) and at the maximum indentation (*d* = 2.7 nm) of the single crystal and penta-twinned nanorod samples.

In previous work^14,16^, the substrate underneath the nanorod was not simulated explicitly. Instead, the positions of several atomic layers at the bottom of the nanorod were frozen, to model the contact with an infinitely hard substrate. This has two effects: first, the actual contact area between the nanorod and the substrate is very small, and therefore some of the load from the AFM tip could be transduced through the nanorod, and deform the substrate underneath–leading to a systematic error in the measured force-distance curves. Second, fixing atoms at the bottom of the sample can influence the plastic deformation mechanism. In order to address these issues, we have simulated the indentation on an explicit silicon substrate, large enough along *x* and *y* directions to avoid periodic boundary effects. As illustrated in Fig. 3, the bonding between gold and silicon atoms creates some misfit dislocations at the Au-Si interface. At the largest indentation depths of 2.7 nm, corresponding to forces around 1 *μ*N in case of the single crystal nanorods, no elastic deformation of the silicon crystal structure under the indented nanorods was observed. In fact, at some positions we even observe a minor shift of ~0.1 nm upwards of the Si atoms at the interface, due to tilting of the sample under load and increased bonding at the interface. In the (110) terminated nanorod, where a dislocation loop annihilated at the bottom of the nanorod, the displaced gold atoms punched a circular area in the silicon substrate (indicated by the black arrow in Fig. 3). In previous work, where the bottom of the gold nanorod had been frozen, the loop had been trapped and stress could not be released in this fashion. However, the basic plastic deformation mechanisms of the three nanorods under load were the same, whether or not an explicit substrate had been simulated. Taking into consideration the substantial additional computational cost of including an explicit substrate, we recommend such an approach should only be taken if in a simulation with an implicit or fixed substrate, a potentially important pathway for stress release was found to be blocked.

### Young’s modulus from Oliver-Pharr method

In order to relate our simulations directly to an experimental measurement, we applied the widely used Oliver-Pharr method^23,24^ to calculate the Young’s moduli of the three gold nanorods from the unloading force-distance curves. For *d* > 2.4 nm, the data points on the unloading curve were fitted by a power law, $F(d)=a{(d-{d}_{0})}^{m}$, where *a* and *d*_0_ were the parameters, and $m=\mathrm{3/2}$, because of the spherical shape of the tip apex. In Fig. 2a, the three fits are shown as dashed curves and a vertical black line indicates $d=2.4$ nm. The Young’s modulus was calculated as

1 $$E=\frac{S\mathrm{(1}-{\nu }^{2})}{2\sqrt{\mathrm{(2}R{d}_{m}-{d}_{m}^{2})}},$$

where $S={\rm{d}}F({d}_{m})/{\rm{d}}d$ is the value of the derivative of the fitted force-distance curve at the maximum indentation depth *d* = *d*_*m*_, and $\nu$, and $R=10$ nm denote the Poisson ratio of the sample, and the radius of the tip apex, respectively.

In order to obtain the Poisson ratio for the single crystal nanorods, we performed a simple elongation test with the Au potential used in this work. The Young’s moduli from the stretching simulation were ${E}_{\mathrm{[100]}}=73.7$ GPa, ${E}_{\mathrm{[110]}}=98.7$ GPa. Poisson’s ratio for stretching in [100] was ${\nu }_{\mathrm{[100]}}=0.417$. In the [110] direction, the ratio is different for different lateral directions, which are $\mathrm{[1}\bar{1}\mathrm{0]}$ and [001] in this case. The results were ${\nu }_{\mathrm{[110],[1}\bar{1}\mathrm{0]}}=0.128$, and ${\nu }_{\mathrm{[110],[001]}}=0.654$. The average of these two values, ${\nu }_{\mathrm{[110]}}=0.391$, was used in calculating the Young’s modulus with equation (1). For the penta-twinned nanorod, the bulk Poisson ratio was used. Following the approach by Wood *et al*.^14^, the ‘contact depth’ was approximated by the actual indentation depth *d*. The diamond tip was simulated as a rigid body, and therefore did not contribute to the reduced modulus of the combined system.

We obtained values of $E\sim 103\pm \mathrm{2,}$ $140\pm 4$ and $108\pm 2$ GPa for the (100) terminated, (110) terminated, and penta-twinned nanorods, respectively. The error of *E* has been estimated from the uncertainty in the slope of the respective fitted unloading curve. All three elastic moduli are higher than the Young’s modulus of gold $E=79$ GPa, and the Young’s moduli of the single crystal nanorods from the nanoindentation simulation are higher than the ones obtained from the stretching simulation. The elastic modulus of the (110) terminated rod is significantly larger than of the (100) terminated rod, which agrees with the experimentally determined elastic moduli of gold along the corresponding crystallographic axes, ${E}_{\mathrm{[110]}}=81$ GPa and ${E}_{\mathrm{[100]}}=42$ GPa. The value obtained for the twinned nanorod is hard to interpret, as here the indentation occurs within the twin plane, in the former [221] direction, before reconstruction.

The calculated elastic moduli agree with the highest values observed in AFM bending experiments on gold nanowires with 50 nm diameter^1^. The same study also found that the Young’s modulus increased with decreasing nanowire diameter, a trend confirmed in many simulations^37^, as well as similar experiments on silver nanowires^38^. However, transient absorption experiments on both pentatwinned and single crystal gold nanorods found their direction dependent elastic constants to be 20–25% smaller than the corresponding values in bulk gold^39^ and this weakening has been attributed to the large number of less tightly bound surface atoms. As previously mentioned, the concepts of quantities such as the elastic modulus are pushed to their limits when applied to nanoscale systems, which may be the reason why different experimental techniques may lead to contradictory results.

We also applied the Oliver-Pharr method to the unloading curves obtained on gold nanorod samples of the same structure, but half the size, studied in previous work^16^. Here the nanorods measured approximately $28\times 13$ nm, the tip radius was 5 nm, and the maximum indentation depth was 1.8 nm. Instead of an explicit substrate, the bottom layers of gold atoms in the rods were fixed to their inital positions. The elastic moduli were found to be $E\sim 82\pm \mathrm{3,}$ $92\pm 5$ and $85\pm 7$ GPa for the (100) terminated, (110) terminated, and penta-twinned nanorods, respectively. It is interesting to note that these values are much smaller than the ones reported earlier, obtained from applying the Hertz model^40^,

2 $$F(d)=\frac{4}{3}\frac{E}{\mathrm{(1}-{\nu }^{2})}\sqrt{R}\,{d}^{\frac{3}{2}},$$

to the initial elastic regime of the indentation curve ($E\sim 200$ GPa), and also smaller than the values obtained on the nanorods measuring $55\times 27$ nm. However, the relative order of the elastic moduli for the different structures is consistent between the two system sizes. The discrepancy between results obtained from two different continuum theories suggests that they may not be applicable at these small scales. In addition, the fit to the initial elastic regime in the loading curves required in the Hertz model is somewhat problematic. Due to the use of a Lennard-Jones potential to describe tip and sample atom interactions, the forces are first attractive in a small distance range before becoming repulsive. Therefore the first derivative of the force at the origin of the distance axis does not vanish, as it would in the Hertz model, where the *F*-*d* curve follows a simple power law, $F\sim {d}^{\mathrm{3/2}}$, for a spherical indenter. This different behaviour at small distances introduces a systematic error in the fit. In the Oliver-Pharr method, the unloading curves are fitted to the same power law, but in a distance range where the Lennard-Jones attractions play a minor part. We therefore conclude the latter method is better suited to connect simulations to experiment.

### Limitations of the simulation

While a significant effort was made to match experimental length scales of both the AFM tip and nanorod samples, and an explicit substrate was included, the following issues need to be discussed: the quasi-static indentation scheme had an indentation rate of 0.5 ms^−1^, several orders of magnitude faster than in experiment. While we found that at each indentation depth, the dislocation systems migrated to their equilibrium positions in a fraction of the 50 ps MD trajectory, we cannot fully exclude the possibility that thermal fluctuations could enable the system to cross energy barriers to further displacement of the dislocations on much longer time scales. The influence of other simulation parameters on the atomistic processes observed during indentation was found to be minor, as discussed in previous work^16^. Our simulation setup is highly idealised: the AFM tip and nanorod samples have a perfect geometry, and the silicon (100) substrate has a perfect Si-dimer surface termination. Real samples could exhibit some surface irregularities affecting dislocation nucleation and propagation^41,42^, and unless the experiments were carried out in UHV conditions, the silicon substrate would be covered in an oxide layer several nanometers thick. Furthermore, depending on the method of nanorod deposition, surfactant molecules (which keep the nanorods from agglomerating in solution) could still be attached to their surfaces. Finally, if the experiment is carried out in ambient conditions, all surfaces are covered in a thin film of water, the thickness of which depends on the relative humidity^43^, and a meniscus would form when the tip approached the sample^44–46^. Depending on the details of the interface region, water molecules could remain trapped between the tip and sample during indentation. All these effects can have a strong contribution to the force-distance curves measured and make it difficult to obtain reproducible data in experiments.

In conclusion, we have used atomistic molecular dynamics to study the indentation of three gold nanorods exhibiting different crystallographic structure and orientation, on a silicon substrate, with a diamond AFM tip. Tip and sample sizes were chosen to match experiments. We observe nucleation of defects and propagation of dislocation systems at indentation depths well below 1 nm. Our simulations show how the structure and orientation of the sample determine the atomistic mechanism of the plastic deformation and stress release, because of the different orientations of the {111} glide systems of the fcc structure with respect to the indentation direction and the free surfaces. The dislocation creation and plastic deformation mechanisms in the 55 nm × 27 nm nanorods are similar to the ones identified previously in nanorods half the size. The forces measured on the two differently oriented single crystal nanorods are of similar magnitude – despite their very different plastic deformation mechanisms – and are larger than the ones obtained on the penta-twinned nanorod of similar diameter, because the latter exposes a corner to the tip apex. Using the Oliver-Pharr method, we determined the Young’s moduli of the (100) terminated, (110) terminated, and penta-twinned nanorods to be $E\sim 103\pm 2,$ $140\pm 4$ and $108\pm 2$ GPa, repectively. These values are larger than the bulk modulus of gold, which is consistent with AFM experiments on gold and silver nanowires. Finally, we could not observe elastic deformation of the silicon substrate underneath the nanorods, even at the maximum applied loads of 1 *μ*N, which validates the approach often taken in simulations, where the substrate is omitted and instead a few atomic layers at the bottom of the sample are kept at fixed positions. However, in case of the (110) terminated nanorods, we observed the ejection of a dislocation loop at the bottom of the rod, deforming the substrate – a pathway to stress release not available if the bottom of the nanorod had been held completely fixed.

### Data availability

Simulation input files and datasets generated during the current study are available from the corresponding author on reasonable request.

## Electronic supplementary material

Supplementary information
